# Evaluation of nutritional and physicochemical characteristics of soy yogurt by *Lactobacillus plantarum* KU985432 and *Saccharomyces boulardii* CNCMI-745

**DOI:** 10.1038/s41598-023-40207-4

**Published:** 2023-08-10

**Authors:** Fathy M. Mehaya, Asmaa I. El-Shazly, Asmaa Negm El-Dein, Mohamed A. Farid

**Affiliations:** 1 Food Technology Department, National Research Centre, Cairo, Egypt; 2 Chemistry of Natural and Microbial Products Department, National Research Centre, Cairo, Egypt

**Keywords:** Biochemistry, Biological techniques, Biotechnology, Microbiology

## Abstract

Nutritional yeast-produced soy yogurt has grown in demand, because of its unique nutritional and health benefits. It has low cholesterol, no lactose, and high levels of protein, probiotic yeast, vitamins, and minerals. In this work, Soymilk (12.5%) was prepared and fermented to produce soy yogurt. Growth curves, probiotic characteristics of *Saccharomyces boulardii* CNCMI-745 and *Lactobacillus plantarum* KU985432 were determined. The nutritional value of both yogurts was evaluated, including viable cell count, protein, vitamin B-complex, sugars, phenolic acids, and fatty acids, mineral content, stability, and storage. Analysis of the physicochemical composition of the yogurts included assessment of titratable acidity, antioxidant potential, viscosity, and moisture content. The probiotic viable count of the produced yogurts met the standards for commercial yogurts. *S. boulardii* CNCMI-745 displayed safety characteristics and high tolerance to heat, acid, and alkaline stress*.* The produced B vitamins increased in both yogurts. The total saturated fatty acids in *Saccharomyces*-yogurt decreased, while the unsaturated fatty acids increased. *Saccharomyces*-yogurt showed high antioxidant activity, phenolic acids, and crude protein content. Both yogurts demonstrated the same tendency for stability during 16 day-storage. In conclusion, using nutritional yeast in the production of soy yogurt increased its nutritional content more than probiotic lactic acid bacteria.

## Introduction

Probiotics are living microorganisms that, when consumed in sufficient amounts, improve the health of the host. Yeasts can function as probiotics in addition to the well-known lactic acid bacteria (LAB)^1^. The field of probiotic and potentially probiotic yeasts has been growing, with the possibility for new probiotic products with novel qualities that are not currently present in bacteria-based probiotics. Probiotic bacteria, particularly LAB and Bifidobacteria, are frequently used to produce healthy fermented foods in order to improve their efficacy and quality^2^. Consuming lactic acid bacteria has a number of health benefits, such as (i) enhancing the immune system, (ii) improving the bioavailability of nutrients, (iii) reducing the symptoms of lactose intolerance, (iv) lowering the prevalence of allergies in susceptible people, and (v) lowering the risk of certain cancers^3^. Among fermented dairy foods, yogurt is still the most convenient source of probiotics for consumers^4^. Due to the rising prevalence of lactose intolerance, and hyperlipidemia caused by fermented dairy products, new vegan sources have been investigated as potential substrates for probiotics^5^. Soy is an excellent substitute for dairy products because of its ability to overcome dairy product constraints^6^. Fermented soy products have been seen in numerous studies to offer a wide range of health benefits as being effective probiotic sources, including decreasing blood cholesterol, anti-diabetic, anti-hypertensive, anti-cardiovascular, and anti-neuroinflammatory properties^4,7^. *S. boulardii* is an exceptional probiotic and biotherapeutic yeast. It can survive in the human gastrointestinal (GI) tract, withstand low pH and bile salts exposures, and does not affect the normal microbiota. It could relieve diseases such as acute diarrhea in children and *Clostridium difficile* associated diarrhea^8^. As a result, it is frequently found in the food and supplement industries. It has been approved by the Food and Drug Administration (FDA) as a safe supplement^9^. However, several studies have been discussed fermenting soymilk to produce soy yogurt, no sufficient data on the use of *S. boulardii* to produce soy yogurt is available. Therefore, our work was designed to provide data on nutritional composition and physicochemical characteristics of soy yogurts produced by *S. boulardii* CNCM I-745 and *L. plantarum* KU985432.

## Materials and methods

### Materials

Daidzein, genistein, Folin–Ciocalteau reagent, and gallic acid were obtained from Sigma Company (St. Louis, MO, USA). Methanol of the HPLC grade was purchased from Fisher Scientific (Hanover Park, Illinois, USA). Other chemicals were of the analytical degree. *Lactobacillus plantarum* KU985432 was previously isolated and identified^10^. *S. boulardii* CNCMI-745 (*Saccharomyces cerevisiae* HANSEN CBS 5926, Perenterol, GmbH, Germany).

### Methods

#### Growth curve of *S. boulardii* CNCMI-745 and *L. plantarum* KU985432

*L. plantarum* KU985432 was cultivated in MRS broth medium, while *S. boulardii* CNCMI-745 was cultivated in potato dextrose broth medium, at 37 °C. The initial concentration of *L. plantarum* KU985432 and *S. boulardii* CNCMI-745 were 0.33 × 10^9^ and 0.53 × 10^7^ CFU/mL, respectively. The samples were gathered at 0, 2, 4, 8, 24, 48, 72, and 96 h and enumerated using the pour plate method^11^. The plates were incubated for 48 h at 37 °C, and the viable cell count of both strains was estimated.

#### Probiotic properties

Stress tolerance tests were characterized for *S. boulardii* CNCMI-745^12^. The stress tolerance response of *S. boulardii* CNCMI-745 evaluated by subjecting live cells to various stress conditions. The culture of *S. boulardii* CNCMI-745, which grew on potato dextrose broth at 37 °C, was adjusted to about 0.6 at OD_600_. After 10-min centrifugation at 6000 rpm, yeast cells were re-suspended in various stress solutions after being washed twice with 20 mL of sterile, pH 7, 0.2 M sodium phosphate buffer. Yeast cells were then sub-cultured in potato dextrose broth and kept at 37 °C for 48 h. Controls grown on nutrient broth for 48 h at 37 °C were regarded as having 100% viability. Experiments were done in duplicate, expressed as mean ± SD and the results were compared to the control.

#### Safety aspects evaluation: Hematolytic activity

The hemolytic activity of yeast cells was used to assess their safety^13^. The technique involved plating actively growing cells onto Columbia-agar enriched with 5% (v/v) animal blood, which was used to measure the production of hemolysin. The plates were incubated aerobically at 37 °C for 24–48 h because anaerobic incubation might interfere with hemolytic activity.

#### Safety aspects evaluation: Histamine production

Decarboxylase agar medium was used to test the production of histamine^14^. Yeast cells were then streaked in duplicate on decarboxylase medium plates with and without histidine (as a control) and incubated for 4 days at 37 °C under aerobic conditions.

#### Production of soy yogurt

Soymilk was prepared according to the pervious method^15^. Soybeans were washed, soaked overnight in distilled water and then grinded with distilled water (1:8 w/v). The slurry was filtered through double-layered cheesecloth to separate insoluble residues. The soymilk was then autoclaved at 121 °C for 15 min. 150 mL of the soymilk was inoculated with 10% of *L. plantarum* KU985432 or *S. boulardii* CNCMI-745 and left unshaken at 40 °C for 48 h. Before inoculation, the probiotic cultures were centrifuged at 4 °C and 5000 rpm for 10 min and washed twice with sterilized distilled water.

#### Acid production

The titratable acidity of both yogurts, which corresponds to the amount of sodium hydroxide (0.05 M) required to titrate a certain amount of the sample to a pH of 8.1, was also determined^16^.

#### Cell viability

The initial and final viable counts of *L. plantarum* KU985432 and *S. boulardii* CNCMI-745 were determined after 48 h of fermentation. Probiotic cultures were inoculated onto modified MRS agar for *L. plantarum* KU985432 and potato dextrose agar for *S. boulardii* CNCMI-745. All plates were incubated for 48 h at 37 °C. The count of viable cells was determined and expressed as CFU/mL^17^.

#### Stability and storage of yogurts

The viable cell count of *L. plantarum* KU985432 and *S. boulardii* CNCMI-745 used in preparing yogurt were monitored periodically for 16 days. Yogurt samples were preserved in the refrigerator. The viable cell count was determined after 48 h incubation by plate count agar^11^.

#### Extraction of soy yogurt

Extracts for total phenolic compounds and antioxidant activity were prepared using methanol. Ten grams from each yogurt sample was mixed with 100 mL methanol and homogenized using the Ultra-Turrax homogenizer. The homogenates were stored at 4 °C for 12 h before being centrifuged at 10,000 rpm for 20 min. The supernatants were recovered and stored at – 20 °C until analysis.

#### ABTS radical cation scavenging assay

The ability of yogurt extracts to scavenge 2,2′-azino-bis(3-ethylbenzothiazoline-6-sulfonic) acid (ABTS) radical cation was performed as described by Re et al.^18^.

#### Determination of total phenolic content

The total phenolic content was determined by Folin-Ciocalteu reagent according to Žilić et al.^19^.

#### Phenolic acids profile by HPLC

The sample (1 g) was hydrolysed with 20 mL of 2 M NaOH for 4 h at room temperature. The pH of the samples was adjusted to 2 with 6 M HCl. Then, phenolic compounds were extracted twice with 50 mL of a 1:1 mixture of ethyl ether and ethyl acetate. The organic phase was separated and evaporated at 45 °C and the samples were redissolved in 2 mL methanol. Chromatographic analysis of phenolic acid and isoflavones were performed by HPLC model 1100 series (Agilent Technologies, CA, USA)^20^.

#### Determination of water-soluble vitamins by HPLC

The extraction solution was made by mixing 50 mL of acetonitrile with 10 mL of glacial acetic acid, and the volume was finally made up to 1000 mL with double distilled water. Each sample (10 g) was weighed and homogenized in a mortar with a pestle before being transferred to a conical flask with 25 mL of extraction solution and kept in a shaking water bath at 70 °C for 40 min. The sample was then chilled, filtered, and the volume was adjusted to 50 mL with extraction solution. After that, the sample was filtered through 0.45 m filter tips, and aliquots of 20 µL from this solution were injected into the HPLC by auto-sampler. The analysis and quantification of vitamins in samples were performed using an Agilent 1100 chromatographic system (Agilent Technologies, CA, USA)^20^**.**

#### Determination of sugars by HPLC-RID

The yogurt samples were extracted with 20 mL of deionized water and sonicated for 30 min. The final volume of solution was completed to 50 mL and filtrated through a 0.45 µm syringe filter. Sugars (glucose, fructose, and sucrose) were determined by Agilent Technologies 1100 series liquid chromatograph equipped with an autosampler, a refractive index detector, and an SCR-101N analytical column. The mobile phase was water with a flow rate of 0.7 mL/min at 40 °C of the oven temperature. The injection loop was optimized for 5 µL. The concentrations of the products were determined from the peak area under the curve^21^.

#### Determination of fatty acid profile by GC–MS

The analysis of fatty acids was carried out using GC–MS system (Agilent Technologies) gas chromatograph (7890B) was equipped with mass spectrometer detector (5977A). The GC was equipped with HP-5MS column (30 m × 0.25 mm internal diameter and 0.25 μm film thickness). The derivatization of fatty acids was performed by 1% sodium methoxide in methanol^22^. Identification of different constituents was determined by comparing the spectrum fragmentation pattern with those stored in Wiley and NIST Mass Spectral Library data.

#### Chemical composition

Moisture, protein (N × 6.25), fats (ether extract), ash, and crude fiber contents were determined according to^23^. Total carbohydrates were calculated by difference. Determination of minerals (Ca, K and Fe) was carried by atomic absorption, while phosphorus was determined by the spectrophotometer series^24^.

## Results

### Growth curve of *L. plantarum* KU985432 and *S. boulardii* CNCMI-745

**Figure 1:**
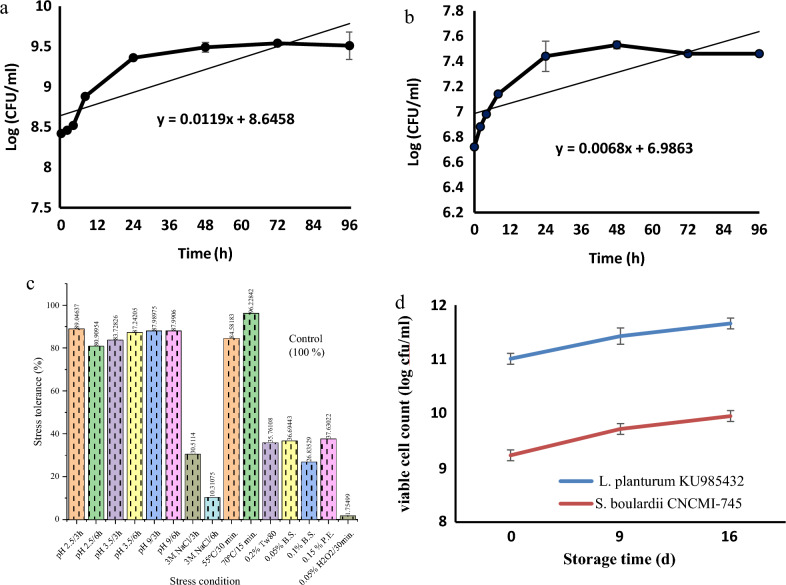
(**a**) Time and log CFU/ml empirical relation of *L. plantarum* KU985432 in MRS medium; (**b**) Time and log CFU/ml empirical relation of *S. boulardii* CNCM I-745 in PDA medium; (**c**) Probiotic properties of *S. boulardii* CNCM I-745; (**d**) The change of *L. plantarum* KU985432 and *S. boulardii* CNCM I-745 viable counts in yogurts samples produced with soy milk during storage.

*L. plantarum* KU985432 and *S. boulardii* CNCMI-745 growth curves are presented in Fig. 1a and b. The relation based on colony counting (Log CFU/mL) (Y-axis) against time (h) (X-axis) of these strains is given by the empirically derived equation Y = 0.0119X + 8.6458 and Y = 0.0068X + 6.9863, respectively. This equation described the growth pattern of the bacterial and yeast cells in MRS and PDA media at pH 6.5 and 37 °C, respectively. From measured growth curves (Fig. 1a,b), the exponential (Log) growth phase of *L. plantarum* KU985432 and *S. boulardii* CNCMI-745 was the same as observed between 8 and 48th h at 37 °C.

### Probiotic properties of *S. boulardii* CNCMI-745

*S. boulardii* CNCMI-745 probiotic characteristics were determined, just as they had been done for *L. plantarum* KU985432 by^10^. As depicted in Fig. 1c, *S*. *boulardii* CNCM I-745 exhibited high heat, acid, and alkaline stress resistance. It survived the exposure to pH 2.5, 3.5, and 9.0 for 3, and 6 h, cell survival ranged from 80 to 89%. It also managed to survive exposure to high temperatures up to 55 °C for 30 min and 70 °C for 15 min, respectively, with survival rates of 84 and 96%. However, the overall resistance to osmotic, surfactant and enzymatic stress was considerably lower, the exposure to 0.05% H_2_O_2_ did not totally kill the cells, and it could survive and regrow again after sub-culturing the cells. Additionally, the safety characteristics were assessed to ensure the safe use of this probiotic yeast in food products. Fortunately, *S. boulardii* CNCMI-745 has no positive histamine production or blood hemolysis in the screening medium. It was a negative histamine producer and γ-hemolytic.

### Production of soy yogurt

Viable cell count of *Lactobacillus*-yogurt increased from 1.2 to 1.7 × 10^8^ CFU/mL (Table 1). The final viable cell count of *S. boulardii* CNCMI-745 in the produced yogurt also increased from 1.475 to 1.795 × 10^8^ CFU/mL. The highest titratable acidity was seen in soymilk fermented with *L. plantarum* KU985432, and the lowest was in *Saccharomyces*-yogurt compared to unfermented soymilk.

**Table 1:** Characteristics of soy yogurt produced by *L. plantarum* KU985432 and *S*. *boularrdii* CNCM I-745.

Soy yogurt	Final pH	Titratable acidity (% lactic acid)	Initial viable count (CFU/ml) × 10^8^	Final viable count (CFU/ml) × 10^8^	Total phenolics (GAE* mg/g)	ABTS** (mgTE***/g)
*Lactobacillus*-yogurt	4.0	0.90	1.275 ± 0.04	1.70 ± 0.35	2.29 ± 0.09	5.86 ± 0.18
*Saccharomyces*-yogurt	4.4	0.29	1.475 ± 0.23	1.795 ± 0.05	3.52 ± 0.14	6.02 ± 0.014
Unfermented soymilk	7.0	0.11	Non-inoculated	Non-inoculated	2.26 ± 0.05	4.45 ± 0.014

*Milligrams of gallic acid equivalent (mg GAE).

**2,2′-azino-bis(3-ethylbenzothiazoline-6-sulfonic) acid (ABTS).

***TE = Trolox equivalent.

### Antioxidant activity

Soymilk fermented with *S. boulardii* CNCMI-745 expressed the highest radical scavenging activity (6.02 mgTE/g) compared to *L. plantarum* KU985432 (5.86 mgTE/g) and unfermented soymilk (4.45 mgTE/g) (Table 1). Total phenolic compounds were found to be highest in Saccharomyces-yogurt (3.52 GAE mg/g) followed by Lactobacillus-yogurt (2.29 GAE mg/g) and unfermented soymilk (2.26 GAE mg/g) (Table 1).

### Phenolic acids and isoflavones contents

The concentration of phenolic acids (Gallic, Protocatechuic, Syringic, Daidzein, and Genistein) increased after 48-h of fermentation (Table 2). Gallic, protocatechuic and syringic acids were not detected in soy milk, but following *S. boulardii* CNCMI-745 and *L. plantarum* KU985432 fermentation, it increased to 12.196 and 11.444, 10.348 and 47.785, 22.541 and 18.422 µg/100 g DW, respectively. Vanillic acid was detected only in *Lactobacillus*-yogurt (24.040 µg/100 g DW). The amount of catechins and *p-*hydroxybenzoic decreased in *Lactobacillus*-yogurt and increased in *Saccharomyces*-yogurt compared to unfermented soymilk. Fermentation of soymilk by *S. boulardii* CNCMI-745 increased catechins and cinnamic acid content more than in *Lactobacillus*-yogurt and unfermented soymilk. Coumaric acid in the unfermented soymilk was not detected in both yogurts after 48 h fermentation. Table 2 illustrates how fermentation with *L. plantarum* KU985432 and *S. boulardii* CNCMI-745 affected the change in isoflavones. Isoflavone aglycones, daidzein, and genistein, increased after 48 h fermentation. Compared to unfermented soy milk, the genistein content of *Lactobacillus*-yogurt and *Saccharomyces*-yogurt increased by 327.69% and 270.31%, respectively, while the daidzein content increased by 296.14% and 271.37%, respectively.

**Table 2:** Changes of phenolic acids in soy yogurt.

Phenolic acid concentration (µg/g DW)	Unfermented soymilk	*Lactobacillus*-yogurt	*Saccharomyces*-yogurt
Gallic	ND*	11.444	12.196
Protocatechuic	ND*	10.348	47.785
*p-*Hydroxybenzoic	131.688	27.489	122.514
Cateachin	183.108	53.300	243.827
Syringic	ND*	22.541	18.422
Vanillic	ND*	24.040	ND*
*p*-Coumaric	114.276	ND*	ND*
Diadzein	186.338	551.830	505.664
Genistein	113.629	372.349	307.147
Cinnamic	7.824	2.110	18.165

*ND not detected.

### Fatty acids profile

The profile of fatty acids is shown in Table 3. After 48 h of fermentation with *L. plantarum* KU985432 or *S. boulardii* CNCMI-745, both content of saturated fatty acids (myristic, margaric, and arachidic acids) and unsaturated fatty acids, including, oleic acid, linoleic acid, α-linolenic acid, and eicosenoic acid of soy milk increased. However, in *Lactobacillus*-yogurt and *Saccharomyces*-yogurt, the content of palmitic, trans-oleic, and cis-linoleic acids was reduced, although stearic acid was the same as in unfermented soymilk. In *Saccharomyces*-yogurt, the total saturated fatty acids decreased from 18.99 to 18.36%, whereas the unsaturated fatty acids increased from 81.01to 81.31%.

**Table 3:** Changes of fatty acids in soy yogurt.

Fatty acid (relative %)	Unfermented Soymilk	*Lactobacillus*-yogurt	*Saccharomyces*-yogurt
Saturated fatty acid			
Myristic acid C14:0	0	0.14	0.1
Palmitic acid C16:0	13.06	12.78	12.2
Margaric acid C17:0	0	0.11	0.1
Stearic acid C18:0	5.5	5.47	5.56
Arachidic acid C20:0	0.43	0.4	0.5
Total	18.99	18.9	18.36
Unsaturated fatty acids			
Palmitoleic acid C16:1	0	0.43	0
Cis-oleic acid C18:1	21.91	22.32	22.19
Trans-oleic acid C18:1	1.28	1.05	1.17
Cis-linoleic acid C18:2	51.9	48.41	51.21
Trans-linoleic acid C18:2	0.34	1.9	ND*
Α-linolenic acid C18:3 N6	5.58	6.64	6.47
Α-linolenic acid C18:3 N3	0	0.28	0.17
Eicosenoic acid C20:1	0	0.03	0.1
Total	81.01	81.06	81.31

*ND not detected.

### Vitamin B-complex

The changes in B vitamin (B1, B2, B6 and B9) in both soy yogurts are shown in Table 4. The thiamine (B1) content of unfermented soy milk was increased from 26.93 to 44.31 and 40.14 µg/g for both *Lactobacillus*-yogurt and *Saccharomyces*-yogurt at 48 h fermentation. Results showed that riboflavin content was increased to 3.13 µg/g in *Lactobacillus*-yogurt, followed by *Saccharomyces*-yogurt (2.28 µg/g), at 48 h fermentation, compared with unfermented soymilk, which contains 1.36 µg/g (Table 4).

**Table 4:** Changes of B vitamins and sugars in fermented soy yogurt.

Soy yogurt	Vitamin concentration (µg/g DW)				Sugar concentration (g/100 g DW)		
	B1	B2	B6	B9	Sucrose	Glucose	Fructose
*Lactobacillus*-yogurt	44.31	3.13	74.34	49.21	1.165	0.447	0.041
*Saccharomyces*-yogurt	40.14	0.28	196.42	149.55	1.326	2.28	0.089
Unfermented soymilk	26.93	1.36	287.84	149.27	3.797	1.140	0.675

The pyridoxine content (B6) and folate content (B9) of soymilk fermented by *L. Plantarum* KU985432 decreased more than *S. boulardii* (Table 4). Compared to unfermented soymilk, the folate level was about the same after fermentation by *S. boulardii* CNCMI-745.

### Sugar profiles

The change in reducing and non-reducing sugars showed an overall downward trend (Table 4). After 48 h of fermentation, the non-reducing sugar sucrose content of soymilk decreased in *Lactobacillus*-yogurt and *Saccharomyces*-yogurt, respectively, from 3.797 to 1.165 and 1.326 g/100g. Within two days of fermentation, the monosaccharides, fructose, and glucose were all nearly depleted. After, 48-h fermentation process, the glucose level of unfermented soy milk reduced from 1.140 to 0.447 and 0.28 g/100 g in *Lactobacillus*-yogurt and *Saccharomyces*-yogurt, respectively. The fructose content of unfermented soymilk decreased as well (Table 4). After, *S. boulardii* CNCMI-745 fermentation, sugars were reduced more than *L. plantarum* KU985432 fermentation.

### Physicochemical composition of soy yogurt

The protein content in both soy yogurts indicated that fermenting soymilk with the two probiotic cultures increased its protein content (Table 5). Results also showed that unfermented soymilk samples had a lower fat content than *Lactobacillus*-yogurt and *Saccharomyces*-yogurt samples. *Lactobacillus*-yogurt and *Saccharomyces*-yogurt samples had almost the same fat contents. A sample of unfermented soy milk contained more ash (0.71%) than samples of *lactobacillus*-yogurt and *Saccharomyces*-yogurt. *Lactobacillus*-yogurt had less carbohydrate content than *Saccharomyces*-yogurt and unfermented soymilk (Table 5). Unfermented soymilk, and *Lactobacillus*-yogurt and *Saccharomyces*-yogurt had nearly the same moisture content of 95.75, 94.32 and 95.48%, respectively. For minerals content, both yogurts had higher minerals content than unfermented soymilk, except for Zn (Table 5). *Lactobacillus*-yogurt had the same content of Zn that was found in unfermented soymilk.

**Table 5:** Physicochemical composition of soy yogurt.

Physicochemical composition in dry weight basis	*Lactobacillus*-yogurt	*Saccharomyces*-yogurt	Unfermented soymilk
Protein (%*)	48.89	49.76	45.85
Fat (%)	30.876	32.733	31.038
Ash (%)	6.830	6.909	7.074
Carbohydrate (%)	10.598	13.404	14.925
Moisture (%)	94.32	95.48	95.75
Viscosity (cP**)	376.5	302	22
Minerals (ppm***)			
Ca	182.065	228.261	141.304
P	538.043	576.087	429.348
Cu	2.391	2.935	1.630
Fe	11.413	12.500	9.239
Mn	4.076	4.076	2.717
Mg	173.913	217.391	146.739
K	1505.435	1880.435	1211.957
Na	152.174	157.609	86.957
Zn	5.978	7.609	5.326

*Measured and expressed as g/100.

**Centipoise.

***Particles per million.

### Stability and storage of soy yogurt

Figure 1d shows the changes in viable counts of *L. plantarum* KU985432 and *S. boulardii* CNCMI-745 in both yogurts. *L. plantarum* KU985432 and *S. boulardii* CNCMI-745 viable counts increased at the same rate until the end of the 16-day storage period at 6 °C.

## Discussion

The field of fungal probiotics is one that is currently evolving. Yeast contains a massive and diversified population of microorganisms, which is drawing and extending the interest of researchers and companies^1^. In this work, *S. boulardii* CNCMI-745 as a probiotic yeast was used to produce soy yogurt, so its probiotic characteristics were evaluated. The same probiotic characteristics were found by Graff et al.^25^. They found that *S. boulardii* can survive at body temperature (37 °C), giving it the distinct benefit of being one of the few yeasts that thrive at human body temperatures. It can also tolerate gastric acid and bile salt. *S. boulardii* CNCMI-745 was also evaluated for its safety and it was negative histamine producer and γ-hemolytic. Similarly reported by Fernández-Pacheco et al.^26^ Cell wall of *S. boulardii* is thicker than that of other yeasts. These cell wall features can explain some of its probiotic effects, such as tolerance to stress produced by fluctuations in regular and simulated gut pH values^8^. We had successfully produced two fermented soy-yogurts using *L. plantarum* KU985432 and *S. boulardii* CNCMI-745. viable cell count of *L. plantarum* KU985432 and *S. boulardii* CNCMI-745 in both yogurts increased. Most LAB prefer to grow in a neutral pH, which is given by soymilk^27^. Similarly reported, the yeast *S. boulardii* growth in fermented soymilk reached a maximum after 48 h of incubation, ranging from 7.57 to 7.87 log CFU/mL^28^. Across many countries, viable counts of yogurt products should be between 10^6^ and 10^9^ CFU/mL. As a result, both soy yogurts prepared in this work meet the commercial yogurt product criteria. *Lactobacillus*-yogurt showed a more acidic pH value than *Saccharomyces*-yogurt. Probiotic bacteria in soymilk produce galactosidase, which converts oligosaccharides (raffinose, stachyose, and sucrose) to lactic acid in varying amounts depending on the strain's galactosidase activity^27^, so pH of soy milk decreased during fermentation as the incubation period progressed. Soymilk fermented with *S. boulardii* CNCMI-745 expressed the highest radical scavenging activity compared to *L. plantarum* KU985432. It was also found that *Saccharomyces*-yogurt had the highest amount of total phenolic compounds followed by *Lactobacillus*-yogurt, compared to unfermented soymilk. So, the potent antioxidant activity of both yogurts may be due to that both yogurts had a higher content of total phenolics, and fatty acids than unfermented soymilk. in coincidence with that result, we found *Saccharomyces*-yogurt contained higher concentrations of phenolic acids like cinnamic acid, protocatechuic acid, gallic acid, *p*-hydroxybenzoic acid, and catechins than *Lactobacillus*-yogurt did. Cinnamic acid and its derivatives, particularly those with the phenolic hydroxyl group, are well-known antioxidants with various health benefits attributed to their high free radical scavenging abilities^29^. Protocatechuic acid, and protocatechuic aldehyde have been shown to have pharmacological effects both in vitro and in vivo, which include antioxidant activity^30^. Increased concentration of gallic acid in fermented yogurts could be du to that hydrolysable tannins of soy can be transformed to gallic acid^31^. Daidzein, and genistein increased also after 48h fermentation. That increment could be due to β-glucosidase which produced during probiotic soymilk fermentation. β-glucosidase hydrolyzes the glucosidic bond of glucosidic daidzin and genistin into their aglycone forms^32^. The extract of Cheonggukjang (traditional Korean fermented soybeans) containing genistein and daidzein had potent antioxidant activity *in vitro*^33^. The increased phenolic acid concentration during fermentation with *L. plantarum* KU985432 or *S. boulardii* CNCMI-745 was most likely due to microbial constitutive enzymes activating insoluble or bound phenolic acids, resulting in phenolic acids liberation^34^. However, the decline in coumaric acid content could be attributed to the formation of *p*-hydroxybenzoic acid due to the degradation processes^35^. In *Saccharomyces*-yogurt, the total saturated fatty acids decreased, whereas the unsaturated fatty acids increased. Twelve or more fatty acids were reportedly found in wine fermented by various species of Saccharomyces at various temperatures and sweet potato fermented by *S. bouldardii* with higher levels of myristic acid, stearic acid, linolenic acid, and Docosahexaenoic acid (DHA, omega-3) in comparison to the control^36^. Solid-state fermentation with *L. casei* increased the omega-3 fatty acids and decreased the saturated fatty acids content of soybean flour^37^. In *Lactobacillus* and *Saccharomyces*-yogurts, the thiamine (B1) content increased. This may be because the two probiotics can synthesize vitamin B1. Similarly reported, the thiamine level of fermented cashew apple juice by *L. acidophilus* was increased significantly throughout the fermentation period^38^. For riboflavin (B2) content, it also increased after fermentation by *L. plantarum* KU985432 and *S. boulardii* CNCMI-745. Similarly, *L. acidophilus* isolated from dairy samples could produce riboflavin^39^. Riboflavin could be synthesized by *Lactobacillus* spp isolated from a traditional sourdough^40^. Moreover, the increase in riboflavin concentration in fermented-probiotic products could be attributed to the riboflavin production pathway in probiotic bacteria^41^. The low level of pyridoxine (B6) and folate (B9) in *Lactobacillus*-yogurt could be ascribed to LAB requiring for their growth^38^. However, the amount of pyridoxine was increased in cash apple juice fermented by *L. casei*^38^. Furthermore, folate (B9) levels increased in fermented potato substrates produced by two *L. sakei* strains increased^42^. According to sugar analysis soy yogurts by HPLC-RID*, S. boulardii* CNCMI-745 fermentation resulted in a greater reduction in sugars than *L. plantarum* KU985432 fermentation. Yeast invertase can convert sucrose into fructose and glucose in samples associated with *S. boulardii*^44^. Reducing sugars are continuously used as fermentation substrates, which causes their content to decrease because probiotics require sugar metabolism for energy to maintain their propagation during fermentation^43,44^. In green tea fermentation with *S. boulardii*, the depletion of sucrose coincided with an accumulation of fructose within one day but not glucose^45^. The physicochemical analysis of our soy yogurts exhibited a similar tendency to the increased protein content with red yeast fermentation of dry distillers' grain^46^. Similarly reported, Sweet potatoes fermented by *S. bouldardii*^36^. It was noted that the protein found in cells was the reason for the increased protein in milk fermented with Bifidobacteria^47^. *Lactobacillus*-yogurt and *Saccharomyces*-yogurt samples had almost the same fat contents, which was higher than other reported soy yogurt samples. Estevez et al. reported that probiotic-produced yogurt had fat contents that ranged from 22.13 to 22.5%^48^. Rahmatuzzaman Rana et al. produced soy yogurt with 20.07% fat content^49^. Both yogurts had higher ash content than other reported samples. Rahmatuzzaman Rana et al. found the ash content of LAB-fermented yogurt was 3.71%^49^. Fermenting soymilk with *L. delbruckii* subspecies *bulgaricus* and *Streptococcus salivarius* subspecies *termophilus* and treating with concentrated soy proteins resulted in yogurts with higher protein and vegetable fat content while lower in ash and anti-nutrients^50^. *Lactobacillus*-yogurt had less carbohydrate content than *Saccharomyces*-yogurt and unfermented soymilk. As a result, *L. plantarum* KU985432 required more carbohydrates for their growth than that required for the growth of *S. boulardii* CNCMI-745*.*Carbohydrates are needed for the survival or even the growth of lactic acid bacteria, which leads to lactic acid production^51^. Both yogurts showed higher moisture content than other reported plain soy yogurt which was 88.14%^49^. The mineral level of the *Saccharomyces*- and *Lactobacillus* yogurt samples was high, which is consistent with reports of commercial plain yogurts that had higher mineral contents than cow liquid yogurt^52^. During 16-day-storage, both probiotic cultures showed the same tendency towards stability. Gul et al. reported that yeast viable counts leveled up till 14 days of storage; however, after this duration, yeast counts in all kefir samples declined^53^.

## Conclusion

The selection of a probiotic strain for human consumption must be based on the use of the product, as well as the probiotic and other biotechnological properties of the strain. This work demonstrated that soymilk fermented with *S. boulardii* CNCMI-745 had a higher nutritional content than soymilk fermented with *L. plantarum* KU985432. Furthermore, soymilk fermented with *S. boulardii* CNCMI-745 had more antioxidant activity than soymilk fermented by *L. plantarum* KU985432. *Lactobacillus*-yogurt, on the other hand, had higher titratable acidity. Both probiotic cultures demonstrated the same tendency for stability during storage. According to this work, *Saccharomyces*-yogurt is not only a source of probiotics but also a potential component in the production of bioactive compounds such as phenolic acids, fatty acids, and B-vitamins such as thiamine, riboflavin, and pyridoxine, as well as improving the bioavailability of key minerals.

## Future recommendations

Further research could include evaluating the production of soy yogurt using a mixed culture approach of probiotic bacteria and yeast. Additionally, the production of diverse flavors of soy yogurt and their sensory evaluation.

## Data Availability

All data used in this study are available on request from the corresponding author.

## Abbreviations

*Lactobacillus*-yogurt: *Lactobacillus plantarum* KU985432-soy yogurt
*Saccharomyces*-yogurt: *Saccharomyces **boulardii* CNCMI-745-soy yogurt
*S.*: *Saccharomyces*
*L.*: *Lactobacillus*
LAB: Lactic acid bacteria
MRS medium: De Man, Rogosa and Sharpe agar medium
PDA medium: Potato dextrose agar medium
CFU: Colony Forming Unit
HPLC: High performance liquid chromatography
HPLC-RID: HPLC refractive index detectors
GC–MS: Gas chromatography mass spectrometry
TE: Trolox equivalent
GAE mg/g: Milligrams of gallic acid equivalent
DW: Dry weight
cP: Centipoise
ppm: Particles per million
GI: Gastrointestinal
FDA: Food and drug administration
